# Association between Tumor Microbiome and Hypoxia across Anatomic Subsites of Head and Neck Cancers

**DOI:** 10.3390/ijms232415531

**Published:** 2022-12-08

**Authors:** Aastha Dhakal, Rituraj Upadhyay, Caroline Wheeler, Rebecca Hoyd, Vidhya Karivedu, Mauricio E. Gamez, Sasha Valentin, Meade Vanputten, Priyanka Bhateja, Marcelo Bonomi, David J. Konieczkowski, Sujith Baliga, Darrion L. Mitchell, John C. Grecula, Dukagjin M. Blakaj, Nicholas C. Denko, Sachin R. Jhawar, Daniel Spakowicz

**Affiliations:** 1 The Ohio State University College of Medicine, Columbus, OH 43210, USA; 2 Department of Radiation Oncology, The Ohio State University Wexner Medical Center, Columbus, OH 43210, USA; 3 Department of Medical Oncology, The Ohio State University Wexner Medical Center, Columbus, OH 43210, USA; 4 Department of Radiation Oncology, Mayo Clinic, Rochester, MN 55901, USA; 5 Department of Dentistry, The Ohio State University Wexner Medical Center, Columbus, OH 43210, USA; 6 Pelotonia Institute for Immuno-Oncology, The Ohio State University Comprehensive Cancer Center—Arthur G. James Cancer Hospital and Richard J. Solove Research Institute, The Ohio State University, Columbus, OH 43210, USA

**Keywords:** hypoxia, radiation therapy, the cancer genome atlas, head and neck cancer

## Abstract

Purpose/Objective(s): Microbiome has been shown to affect tumorigenesis by promoting inflammation. However, the association between the upper aerodigestive microbiome and head and neck squamous cell carcinoma (HNSCC) is not well established. Hypoxia is a modifiable factor associated with poor radiation response. Our study analyzed the HNSCC tumor samples from The Cancer Genome Atlas (TCGA) to investigate the relationship between different HNSCC tumor subsites, hypoxia, and local tumor microbiome composition. Results: A total of 357 patients were included [Oral cavity (OC) = 226, Oropharynx (OPx) = 53, and Larynx/Hypopharynx (LHPx) = 78], of which 12.8%, 71.7%, and 10.3%, respectively, were HPV positive. The mean (SD) hypoxia scores were 30.18 (11.10), 24.31 (14.13), and 29.53 (12.61) in OC, OPx, and LHPx tumors, respectively, with higher values indicating greater hypoxia. The hypoxia score was significantly higher for OC tumors compared to OPx (*p* = 0.044) and LHPx (*p* = 0.002). There was no significant correlation between hypoxia and HPV status. *Pseudomonas* sp. in OC, *Actinomyces* sp. and *Sulfurimonas* sp. in OPx, and *Filifactor*, *Pseudomonas* and *Actinomyces* sp. in LHPx had the strongest association with the hypoxia score. Materials/Methods: Tumor RNAseq samples from TCGA were processed, and the R package “tmesig” was used to calculate gene expression signature, including the Buffa hypoxia (BH) score, a validated hypoxia signature using 52 hypoxia-regulated genes. Microbe relative abundances were modeled with primary tumor location and a high vs. low tertile BH score applying a gamma-distributed generalized linear regression using the “stats” package in R, with adjusted *p*-value < 0.05 considered significant. Conclusions: In our study, oral cavity tumors were found to be more hypoxic compared to other head and neck subsites, which could potentially contribute to their radiation resistance. For each subsite, distinct microbial populations were over-represented in hypoxic tumors in a subsite-specific manner. Further studies focusing on an association between microbiome, hypoxia, and patient outcomes are warranted.

## 1. Introduction

Head and neck squamous cell carcinoma (HNSCC) is the sixth most common cancer worldwide, and chemoradiotherapy (CRT) is an integral component in their management [1]. Radiation outcomes have been associated with features of the tumor microenvironment, including hypoxia. HNSCC is the most hypoxic among various cancers found in The Cancer Genome Atlas (TCGA) dataset [2]. Tumor hypoxia has been shown to be a biomarker for an aggressive phenotype of head and neck cancers [3]. In fact, the cellular response to conventional radiation techniques depends on the presence of oxygen because the DNA damage affected by low-LET photon radiation requires intratumor oxygen to generate radiolytic hydroxyl radicals, which are the ultimate effectors of radiation-induced DNA damage and tumor cell kill. As such, anaerobic cells in vitro require up to 2.5 times higher radiation doses to achieve the same level of killing as oxygenated cells [3,4]. Furthermore, in clinical practice, hypoxia has been shown to be an independent prognostic factor for treatment outcomes [5]. These observations raise the question of whether a better understanding of the contributors to and consequences of tumor hypoxia could improve patient selection and treatment outcomes.

The microbiome has been shown to affect the tumor microenvironment (TME) by promoting inflammation and producing carcinogenic metabolites [6,7,8,9]. Several microbes have been associated with specific cancers; for example, *Helicobacter pylori* with gastric cancer and *Streptococcus bovis* with colon cancer [9]. The microbiome also affects the immune environment; for example, certain bacterial genotoxins induce DNA damage, resulting in chronic inflammation [10,11]. The interplay between viral pathogenesis and the microbiome has been demonstrated in other tumor sites such as the cervix. Human papillomavirus (HPV) infection is causally related to invasive cervical cancers [12]. Women with early in situ cervical cancers have a higher prevalence of high-diversity *Lactobacillus* spp. depleted vaginal microbiomes [13]. Specific bacteria might play an active role in altering the vaginal microbiome and hastening the progression from cervical intra-epithelial neoplasms to invasive cervical cancers [14]. The relationship between the upper aerodigestive tract microbiome and HNSCC is less well-established. Recent data suggest, however, an association between the oral microbiome and HNSCC [9]. The oral cavity is home to a diverse set of microbes, including viruses, fungi, protozoa, archaea, and bacteria [9]. In a study comparing paired normal and tumor resection specimens from 121 HNSCC patients, tumor tissue tended to have less *Actinomyces* and more *Parvimonas* compared to normal tissue, and the difference increased with higher T-stage [15]. Oral cavity squamous cell carcinoma specifically has more anaerobes compared to normal mucosa [16], which could affect response to radiotherapy (RT) in these tumors due to reduced radiation induced DNA damage in the absence of oxygen. Tumor bacteria and fungi affect responses to immunotherapy, RT, and chemotherapy [17]. By evaluating the microbiome composition found in HNSCC tumor microenvironments, we could potentially modify the environment to augment responses to RT and CRT, and thus improve patient outcomes.

Some factors associated with a poor RT response in HNSCC include HPV-negative status and hypoxia [18]. Oral cavity cancer has among the worst clinical outcomes of upper aerodigestive tract tumors and a worse response to primary treatment with RT compared to surgical series [5,19], leading to it being primarily considered a surgical disease. The differences in the tumor microbiome of radiosensitive HNSCC tumors compared to radio-resistant subsites have not been rigorously investigated. Furthermore, the interplay between the microbiome and local hypoxia has not been reported for HNSCC. It seems plausible that anaerobic microbes may find a hypoxic environment more favorable than a well-oxygenated environment. The purpose of our study was to analyze the relationship between different HNSCC tumor subsites, hypoxia, and the local tumor microbiome in HNSCC using data from The Cancer Genome Atlas (TCGA).

## 2. Results

A total of 357 patients were included [Oral cavity (OC) = 226, Oropharynx (OPx) = 53, and Larynx/Hypopharynx (LHPx) = 78], out of which HPV was present in 12.8%, 71.7%, and 10.3% patients, respectively, in OC, OPx, and LHPx tumors (Table 1).

### 2.1. Hypoxia Scores

**Figure 1:**
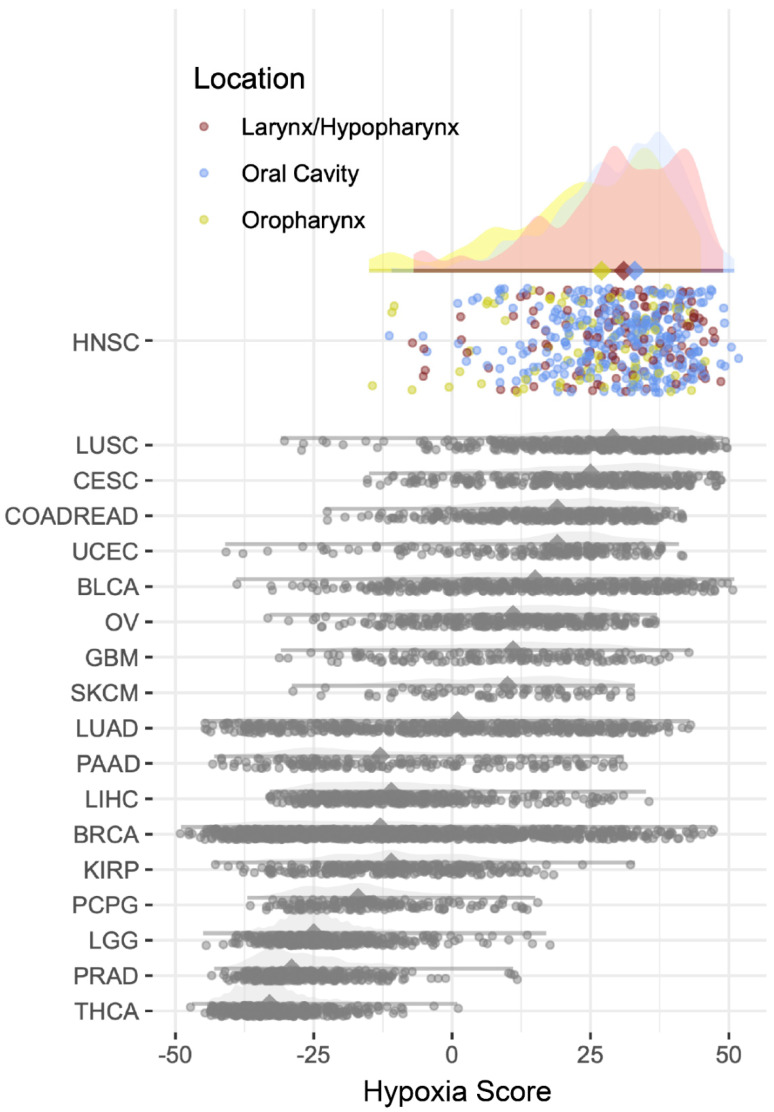
Graph demonstrating that head and neck squamous cancers (HNSC) have the highest hypoxia signature score among the 19 cancers found in The Cancer Genome Atlas (TCGA) dataset. HNSC tumours are further stratified by the subsite (oral cavity, oropharynx, and larynx/hypopharynx). (TCGA abbreviations can be found at https://gdc.cancer.gov/resources-tcga-users/tcga-code-tables/tcga-study-abbreviations, accessed on 20 October 2022).

On comparing the hypoxia scores among all types of cancers in the TCGA dataset of over 12,000 samples, higher scores were seen in head and neck cancers (Figure 1). Stratification by anatomic subsites showed overlapping high hypoxia scores for all head and neck cancers. The mean (SD) hypoxia score for OC was 30.18 (11.10), for OPx 24.31 (14.13), and for LPHx 29.53 (12.61) (Table 1), where a higher positive value of the hypoxia score indicates greater hypoxia. The hypoxia score was significantly higher for OC tumors compared to OPx (*p* = 0.044) and LHPx (*p* = 0.002), indicating greater hypoxia in the oral cavity compared to the other sites. There was no significant difference in the hypoxia scores between OPx and LHPx tumors (*p* = 0.625). There was no significant association between hypoxia and HPV status.

### 2.2. Microbiome of the HNSCC According to Anatomic Subsites and Relationship with Hypoxia Scores

**Figure 2:**
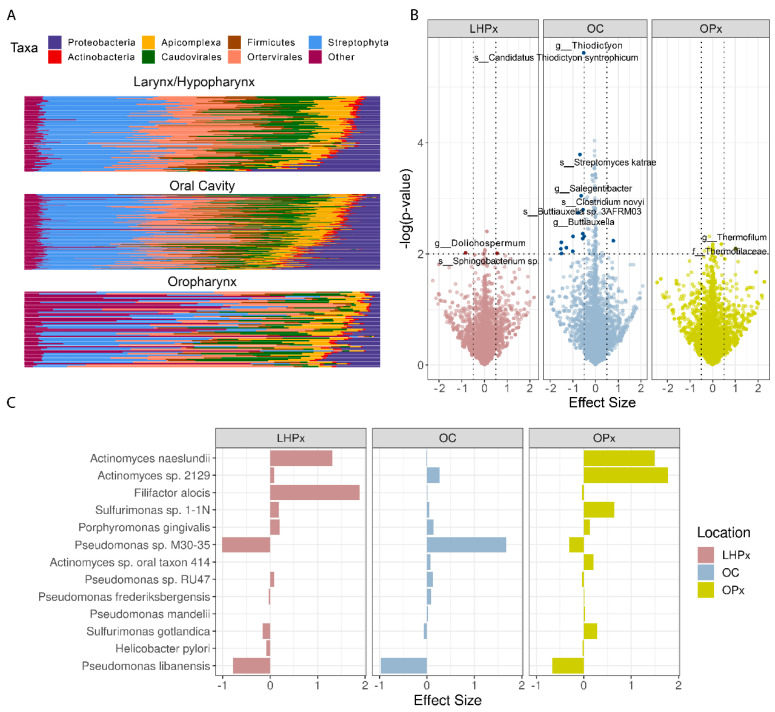
(**A**) Stacked bar plot showing the relative abundance of phyla in each sample stratified by subsite (oral cavity, oropharynx, and larynx/hypopharynx), and ordered by presence of proteobacteria. (**B**) Microbes significantly associated with low (left) or high (right) hypoxia score. The OC has the largest number of significantly associated microbes and with the lowest *p*-values. (**C**) Select microbes strongly associated with the hypoxia score.

The hypoxia expression signature was significantly associated with the differential abundance of 413, 206, and 130 different microbial species phyla in the OC, OPx, and LHPx, respectively. The difference in the number of enriched microbiota reveals the variation in the microbiome of different HNSCC subsites (Figure 2A). Examining the composition of microbiomes for the three tumor subsites showed that the microbe communities were heterogeneous across the three sites, dominated by bacteria with variable abundances of *Ortevirales* viruses (Figure 2A). When analyzing the relationship between microbes and hypoxia, oral cavity tumors were revealed to have microbes associated with greater hypoxia compared to the other two sites (Figure 2B). Anaerobic organisms were found to have the strongest association with hypoxia scores. Specifically, *Pseudomonas* sp. in OC, *Actinomyces* sp. and *Sulfurimonas* sp. in OPx, and *Filifactor, Pseudomonas,* and *Actinomyces* sp. in LHPx showed the strongest associations (Figure 2C); *p*-value < 0.05 for all microbial association. Notably, *Pseudomonas* sp. had the most positive effect size of association with hypoxia score in oral cavity tumors, while a negative association in the other two sites (Figure 2C).

There are several limitations of this study, the most pertinent of which is the retrospective design using the heterogenous population available in the TCGA dataset. The hypoxia expression signature, while validated, is an approximation of the tumor microenvironment. Due to limited clinical and survival data in the TCGA, we were unable to investigate the association of clinical outcomes like radiation treatment with the hypoxia score or the tumor microbiome.

## 3. Methods

We analyzed gene expression in the tumor samples from the TCGA dataset. Hypoxia in the tissue samples was quantified using the Buffa hypoxia signature score, which is a validated gene signature and is calculated from the mean expression of a log of 52 well-characterized hypoxia-regulated genes that were consistently expressed in experiments using HNSCCs, breast, and lung cancer cells and were found to be highly prognostic for survival [20]. The TCGA RNA-seq samples were processed through the exogenous sequencing in tumors and immune cells (exotic) pipeline to identify and count exogenous sequences, filter contaminants, and normalize expression values [21]. Hypoxia score was calculated as described by Buffa et al., 2010 [20], implemented in the R package tmesig [22], following:

$$x_{ij}<med\left(X_{i}\right),\;h_{ij}=-1$$

$$x_{ij}\;\geq med\left(X_{i}\right),\;h_{ij}=1$$

$$H_{j}=\overset{52}{\underset{i=1}{\sum }}h_{ij}$$

where the median value is calculated for all *i* genes and *j* samples. Each expression value is compared to the median to assign a per-gene binary hypoxia score, *h_ij_*. Observations above the median are assigned a positive one, and observations below the median a negative one. The *h_ij_* are then summed over genes to calculate a sample hypoxia score, *H_j_*.

The association of microbes with hypoxic tumors was estimated using logistic regression, fitting the formula:

(1) $$B_{j}=\beta_{0}+\beta_{1}M_{j,\;k}+\varepsilon_{j}\;$$

or

(2) $$B_{j}=\beta_{0}+\beta_{1}M_{j,\;k}+\beta_{2}V_{j}+\beta_{3}M_{j,\;k}V_{j}+\varepsilon_{j}$$

where binarized hypoxia scores (*B_j_*) for each sample, *j*, were assigned to samples in the top and bottom tertiles of the hypoxia score distribution. These values were then treated as the response variable of a model that included predictors for the microbe relative abundance, *M_j,k_*, for each sample, *j*, and microbe, *k*. The HPV status was assessed by including an interaction term for HPV status, *Vj*, binarized as positive if >5 HPV reads were identified in the RNAseq data. Models were constructed using the {stat} package in R [23]. All continuous data were represented as mean and standard deviation (SD). An adjusted [24] *p*-value of <0.05 was considered significant. The code to reproduce all analyses and figures is available at https://github.com/spakowiczlab/exohnsc, accessed on 20 October 2022.

## 4. Conclusions

In this study, we were able to identify an association between specific microbes and relative hypoxia in different HNSCC tumor subsites. Among the subsites, the oral cavity was found to be more hypoxic, which suggests a possible mechanism for radio-resistance of oral cavity tumors. Additional studies are needed to validate our results in datasets with more complete clinical information. Association between certain species such as anaerobes and hypoxia is worth exploring in more detail on other larger patient databases. This will help to establish if there is a link between the hypoxia expression signature, the tumor specific microbiome, and patient outcomes. Further investigation is required to determine if this is a causal relationship and whether modification of the microbiome in the TME can lead to reduced hypoxia and improved radiosensitivity and outcomes. This work will serve as the basis for pre-clinical and early phase clinical trials to assess the manipulation of the tumor microbiome on outcomes in HNSCC.

## Figures and Tables

**Table 1:** The Cancer Genome Atlas (TCGA) HNSC cohort demographics stratified by primary site.

	Oral Cavity	Oropharynx	Larynx/ Hypopharynx	*p*
**Total “n”**	226	53	78	
**Age (mean (SD))**	61.25 (13.27)	58.00 (11.80)	63.11 (9.34)	0.067
**Sex = male (%)**	153 (67.7)	44 (83.0)	61 (78.2)	0.034
**Hypoxia Score (mean (SD))**	30.18(11.10)	24.31 (14.13)	29.53 (12.61)	0.004
**HPV positive (%)**	29 (12.8)	38 (71.7)	8 (10.3)	<0.001

## Data Availability

All data and analysis is available on https://github.com/spakowiczlab/exohnsc, accessed on 20 October 2022.
